# A High Handed Outrage

**Published:** 1899-03

**Authors:** 


A High Handed Outrage.—The latest phase of the "Quarantine War," an account of which we publish elsewhere (from *Gaillard's Medical Journal*) is the indictment and arrest of Dr. Souchon and Dr. Conke, on the charge of manslaughter. The following is from the Associated Press dispatches, clipped from the *Statesman*. That any grand jury could be found to countenance a charge so far fetched and absurd, and so evidently a personal matter, is one of the strange things that cannot be explained:

New Orleans, March 13.—President Edmund Souchon, of the State Board of Health, and Dr. Quitman Conke, of the city board, have been indicted for manslaughter in East Feliciana, in having caused a death in that parish through the introduction of yellow fever from New Orleans. The indictment is the result of charges of Dr. J. MacKowne, who claimed that the doctors, who held that yellow fever was not infectious as they supposed, and that they declined to notify the county parishoners of the existence of alleged fever. The warrants were served today, and Drs. Souchon and Conke were taken to East Feliciana.

The specific charge made is that by failing to promptly announce the first case of yellow fever last summer they prevented quarantine, and thereby permitted fever to enter the city and cause several deaths.

---

The State Medical Association's Bill to regulate the practice of medicine (the Wilson bill) was introduced in the lower house of the Legislature, now in session, as reported in our last issue, and was referred to the Committee on Public Health, of which Dr. Oliver is chairman. This bill provides for three boards, one for each "school," and a medical council, (see our February number). This committee referred the bill to a subcommittee consisting of

Drs. Shelburne, McDonald and Looney. The subcommittee reported favorably on a *substitute bill* which they themselves framed, which is substantially the same as the Wilson bill, minus the medical council, to which the "other fellows"—the homos and other quacks —objected, except that it unites the "schools" in one board of nine members. It also divides the State into four districts, and requires that the board shall meet in each district once each year. The solid board is to consist of nine members so arranged that "no one school shall have a majority of the whole." *Hash!*

---

THE JAPANESE believe in sanitation. The town of Teukcham, on the island of Formosa (taken from the Chinese), has a population of 40,000, with a high death rate. It is situated in a swamp, and cannot be drained. The government has caused the inhabitants to pull up, root and branch, and move to a higher site, some miles off, giving to each householder the same size lot as he had at home—and as the new town was laid off somewhat after the pattern of the old one, except that the streets are wider—a location corresponding to the one he vacated. The government put in sewers, water and light, etc., at public cost.

I wish we had a Japanese governor and legislature in Texas.

---

WHAT SANITATION WILL DO.—The deaths in Havana in February, 1899, were just 51 per cent. less than in February, 1898

